# Internal factors related to self-management among type 2 diabetes patients during the COVID-19 pandemic as humanitarian emergencies: a scoping review protocol

**DOI:** 10.1186/s13643-025-03001-z

**Published:** 2025-12-30

**Authors:** Rie Yamada, Satoshi Kondo, Kuangzhe Xu, Satoshi Okazaki

**Affiliations:** 1 Department of Adult Nursing, Faculty of Medicine, Academic Assembly, University of Toyama, 2630 Sugitani, Toyama-shi, Toyama-ken 930-0194 Japan; 2 Department of Medical Education, Graduate School of Medicine, University of Toyama, 2630 Sugitani, Toyama-shi, Toyama-ken 930-0194 Japan; 3 Center for Medical Education and Career Development, Graduate School of Medicine, University of Toyama, 2630 Sugitani, Toyama-shi, Toyama-ken 930-0194 Japan; 4 Institute for Promotion of Higher Education, Hirosaki University, 1 Bunkyo-Cho,, Hirosaki-shi, Aomori-ken 036-8560 Japan; 5 Department of Intelligent Robotics, Toyama Prefectural University, 5180 Kurokawa, Imizu-shi, Toyama-ken 939-0398 Japan

**Keywords:** COVID-19 pandemic, Type 2 diabetes, Self-management, Internal factors, Scoping review

## Abstract

**Introduction:**

The prevalence and mortality rates of type 2 diabetes mellitus (T2DM) are increasing, making it a significant public health concern. Effective self-management of T2DM requires external factors, such as medical interventions and social support, and internal factors, including self-efficacy. However, during humanitarian emergencies, such as the COVID-19 pandemic, healthcare system disruptions limit external factors, making internal factors even more critical. Nonetheless, existing reviews have primarily focused on external factors and clinical outcomes during the pandemic, with no comprehensive review examining internal factors. Therefore, this review aims to systematically synthesize evidence on the internal factors supporting T2DM self-management during the COVID-19 pandemic and identify research gaps.

**Methods:**

This scoping review follows the Joanna Briggs Institute guidelines and is conducted according to the PRISMA-P framework. A comprehensive search strategy will be employed to systematically explore multiple databases, including PubMed (MEDLINE), CINAHL, Web of Science, Scopus, ERIC, PsycINFO, and the Cochrane Library, as well as gray literature platforms, such as Google, Google Scholar, and Semantic Scholar. The search will include both published and gray literature without language restrictions. Studies that utilize quantitative and qualitative methodologies will also be included. Two independent reviewers will screen titles, abstracts, and full texts for eligibility, and discrepancies will be resolved through discussion or consultation with a third reviewer. The outcomes related to the internal factors contributing to the improvement of self-management among T2DM patients will be extracted and summarized. The findings will be presented descriptively and in tabular form, emphasizing key insights and identifying research gaps in the literature.

**Discussion:**

The findings will be actively disseminated through academic conferences and peer-reviewed journals to provide valuable insights into the internal factors that contribute to the improvement of self-management in T2DM patients. These results are expected to contribute to the development of innovative strategies for sustaining and improving self-management in T2DM patients during future humanitarian emergencies and major public health challenges.

**Supplementary Information:**

The online version contains supplementary material available at 10.1186/s13643-025-03001-z.

## Background

Diabetes is a metabolic disorder characterized by chronic hyperglycemia and has been referred to as “a pandemic of unprecedented magnitude” [1]. As of 2021, approximately 529 million people worldwide were living with diabetes, and this number is projected to exceed 1.31 billion by 2050 [2]. Among the top 10 global causes of death, diabetes claims one life every 5 s due to related complications [1, 3].

Notably, type 2 diabetes mellitus (T2DM), which accounts for approximately 95% of all diabetes cases [2], is associated with a higher risk of macrovascular diseases (e.g., myocardial infarction and cerebrovascular diseases) and microvascular complications (e.g., retinopathy, chronic kidney disease, and neuropathy) [4]. T2DM increases the risk of cancer [5], mild cognitive impairment, and dementia [6]. More than half of T2DM patients experience anxiety, depression [7, 8], and diabetes-related distress [9, 10]. Thus, with its predisposition to various complications, T2DM greatly increases medical expenses [4] and has been recognized as a critical public health challenge in the twenty-first century [1].

To address this critical challenge, the treatment of T2DM must focus on the effective management of blood glucose and glycated hemoglobin (HbA1c) levels to prevent complications [4]. Blood glucose management is closely linked to diabetes self-management [11], which involves daily activities aimed at scientifically and rationally controlling blood glucose levels to prevent complications [12]. T2DM patients must stabilize their blood glucose levels to prevent complications and lead a life tailored to their individual needs.

The self-management of T2DM is not directly influenced by environmental factors such as socioeconomic status or cultural background. Instead, it is shaped by both internal and external factors, ultimately leading to changes in clinical outcomes [13]. Generally, internal factors are a broad concept that encompasses a wide range of personal characteristics, abilities, beliefs, and attitudes. Among these broad concepts, the psychological and cognitive attributes of patients with T2DM have been highlighted in recent studies as key factors that directly support the success of self-management. Indeed, motivation, knowledge [14], coping skills [15], problem-solving abilities [16], and self-efficacy [17] have been consistently reported as crucial internal factors that govern T2DM self-management. Based on these findings, we operationally define internal factors related to T2DM self-management as “the patient’s own psychological and cognitive factors that directly support T2DM self-management.”

Conversely, external determinants include interprofessional collaboration among healthcare professionals [18], structured educational interventions [19, 20], and social support from family and friends [21].

However, unpredictable humanitarian emergencies such as pandemics, natural disasters, and security crises often profoundly impact healthcare systems. This, in turn, significantly limits the external support essential for self-management. These limitations tend to deprioritize the care of noncommunicable diseases, such as diabetes [22]. For example, during wartime, the treatment rate for T2DM decreased by approximately 85% [23], and access to medications and medical supplies was severely restricted during hurricanes [24]. Similarly, during the recent COVID-19 pandemic, shortages in insulin supply, reduced medical support, and decreased access to healthcare have been reported [25, 26]. In addition, routine examinations and self-monitoring markedly declined, with HbA1c testing reduced by up to 77% [27], and self-monitoring by 23% [28]. Reductions in face-to-face interactions due to lockdowns and social distancing have weakened social support [29], with approximately 80% of T2DM patients experiencing negative impacts on their self-management [30]. These circumstances further contributed to decreased physical activity, unhealthy dietary habits, and weight gain [25, 28, 31, 32], as well as increased loneliness, anxiety, and depression [33–36].

The limitations of external resources imposed by crises substantially hinder the self-management of T2DM patients. Therefore, in situations where external support is restricted, internal factors, which represent the personal characteristics and abilities of T2DM patients [14], may play a critical role in adapting to challenging environments and reconstructing their lives.

During the COVID-19 pandemic, approximately 20% of the patients positively perceived the impact of the pandemic [30], roughly 40% consumed healthier foods [37], and 97% consumed freshly prepared meals at home [28]. Furthermore, some patients pay more attention to medication adherence than usual [34], continue regular physical activity and have frequent medical visits [30], and show slight improvement in glycemic control [38]. These findings suggest that even in situations where external factors are severely limited due to health system disruptions, some T2DM patients can utilize internal factors to maintain or improve their self-management. Furthermore, internal factors of self-management are likely to hold significant importance in stabilizing blood glucose levels and preventing complications.

Existing reviews on T2DM self-management during the COVID-19 pandemic have primarily focused on barriers such as lockdowns [26, 39] and healthcare restrictions [40–42], as well as on the deterioration of glycemic control [38, 43, 44]. Additionally, reviews of the support for T2DM self-management during the pandemic have demonstrated the effectiveness of mobile health applications [45, 46] and telehealth [47, 48]. However, these findings have mainly focused on limitations, utilization of external resources, and clinical outcomes. Self-efficacy and other internal factors serve as the foundation of self-management and are crucial for maintaining and improving self-management even in situations where external support is limited [49]. However, a comprehensive review of the internal factors contributing to the improvement of self-management among T2DM patients during the pandemic has not been conducted and remains insufficiently explored.

We focus on the recent COVID-19 pandemic as a critical example of an unforeseen public health emergency. This pandemic has exacerbated the severity and mortality of T2DM [1] and continues to pose challenges owing to the potential resurgence of new variants [50]. Against this backdrop, this review aimed to comprehensively synthesize the existing literature on the internal factors that enable T2DM patients to improve self-management during the COVID-19 pandemic and to identify knowledge and methodological gaps. Specifically, by systematically examining the internal factors that support T2DM self-management, which have yet to be fully explored, this review aimed to establish an empirical foundation for designing sustainable and effective interventions, even in situations where external factors are limited. This is expected to contribute to breaking the vicious cycle in which T2DM has become an increasingly severe public health emergency. Furthermore, this study provides critical insights into stabilizing blood glucose levels, reducing complications and mortality risks, and addressing diabetes as an ongoing public health challenge.

## Methods

Unlike systematic reviews, scoping reviews provide a comprehensive mapping of knowledge by incorporating diverse sources of information regardless of the study design or quality. This method is particularly suitable for exploring emerging or underexplored research topics, defining foundational concepts, and rapidly synthesizing existing evidence. It is also instrumental in identifying research gaps and setting the stage for future investigation [51]. In this protocol, we employ the scoping review methodology to systematically investigate the understudied area of internal factors that facilitate the improvement of self-management among T2DM patients during the COVID-19 pandemic.

The methodology follows a six-stage framework based on Arksey and O’Malley [51] and incorporates the guidelines provided by the Joanna Briggs Institute [52]. Specifically, we will (1) identify the research questions and eligibility criteria; (2) find relevant studies; (3) perform study selection; (4) chart the data; (5) collect, summarize, and report the results; and (6) conclude with consultation. This scoping review will be reported in accordance with the Preferred Reporting Items for Systematic Reviews and Meta-Analyses Protocols (PRISMA-P) [53], specifically the PRISMA extension for a scoping review (PRISMA-ScR) checklist will be followed to report this study [54]. We plan to conduct this scoping review from February 1, 2026, to August 1, 2026. This protocol is registered in the Open Science Framework (https://osf.io/8mhvt/).

### Step 1: identify the research questions and eligibility criteria

Using the population/conceptual/context framework outlined by the Joanna Briggs Institute [52], we established detailed inclusion and exclusion criteria to ensure a structured and comprehensive approach to the review.

- Population: The population includes patients with type 2 diabetes. Patients with type 1 diabetes mellitus, steroid-induced diabetes, or prediabetes will be excluded. Studies where data on patients with type 2 diabetes cannot be separated from those on other patients with diabetes but include over 80% of patients with type 2 diabetes. Internal factors, such as self-efficacy, have a stronger influence on self-management among T2DM patients than age [55]. Therefore, this review will not impose any age restrictions on T2DM patients.
- Concept: This concept focuses on internal factors that contribute to the improvement of self-management among T2DM patients. Diabetes self-management refers to daily activities aimed at scientifically and rationally controlling blood glucose levels and preventing complications [12]. These include appropriate dietary management, regular physical activity, pharmacotherapy (e.g., oral hypoglycemic agents and insulin injections), and self-monitoring of blood glucose levels [12]. Additionally, the related concepts of self-care and self-regulation were considered [56]. Self-care refers to everyday activities aimed at maintaining health and enhancing quality of life (QOL), whereas self-regulation denotes the capacity to control and adjust emotions, thoughts, and behaviors in pursuit of personal goals [56]. A preliminary literature search revealed that some studies describe the self-management behaviors of patients with T2DM using these alternative terms. Therefore, to align with this review’s objective of comprehensively mapping the existing literature, self-care and self-regulation were also included as related concepts.

We specified indicators or outcomes as the objective markers used to evaluate self-management. These markers comprise biological data, such as HbA1c and self-monitored blood glucose [57], and behavioral data, including dietary habits, medication adherence, physical activity, and insulin management [58]. In contrast, we characterized measurement tools or scales as standardized scales that assess the psychological or cognitive constructs influencing this self-management, such as self-efficacy and quality of life (QOL). Internal factors associated with self-management among T2DM patients include resilience, self-efficacy, diabetes distress, meaningfulness of life, and self-confidence [17]. Additionally, health literacy [59], stress management behavior and coping skills [15], problem-solving ability [16], motivation [60], decision-making ability [61], self-control [13], illness beliefs, and health belief [62] have also been reported as internal factors that influence self-management in T2DM patients. Therefore, these factors will be included in this review. External factors such as social support and healthcare provider interventions related to self-management among T2DM patients will be excluded.

(c) Context: This context focuses on the COVID-19 pandemic, specifically from March 11, 2020, when the World Health Organization (WHO) declared COVID-19 a Public Health Emergency of International Concern [63], to May 5, 2023, when the emergency status was lifted [64]. Studies unrelated to the pandemic are excluded, even within this period.
(d) Type of source: The review will consider both peer-reviewed primary studies and gray literature, including non-commercially published reports and documents that have not undergone formal peer review. No restrictions will be applied regarding the study design, including experimental studies, observational research, qualitative studies, or systematic reviews. Additionally, no limitations will be imposed on the languages of the included studies.

This scoping review intends to address the following questions:

1. What evidence has been reported regarding the internal factors contributing to the improvement of self-management among T2DM patients during the COVID-19 pandemic?
2. What measurement tools or scales have been used to evaluate the improvement of self-management in T2DM patients?
3. What outcomes or indicators have been used to evaluate the improvement of self-management in T2DM patients?

### Step 2: identify relevant studies

The literature search will utilize multiple databases, including PubMed (MEDLINE), CINAHL, Education Resources Information Center (ERIC), Web of Science, PsycINFO, Cochrane Library, and Scopus. Additionally, gray literature will be identified through searches conducted on platforms such as Google, Google Scholar, and Semantic Scholar.

The search strategy is tailored to each database by employing comprehensive keyword combinations, Medical Subject Headings (MeSH), and subject terms. These terms are structured to align with the population (T2DM patients), concept (internal factors that contribute to the improvement of self-management), and context (during the COVID-19 pandemic), combined with the Boolean operators “AND” and “OR.” The initial PubMed (MEDLINE) search strategy, designed to ensure comprehensive coverage, is outlined in Table 1 (Additional file 1). This initial search was conducted by the lead author on October 15, 2025, under the guidance of a medical librarian specializing in systematic reviews and database searches.

Screening gray literature is time-consuming, and the likelihood of finding highly relevant articles is low [65]. Therefore, in this review, we will screen the top 100 results from each platform (e.g., Google, Google Scholar), ranked by default relevance. These results are regarded as the most relevant [65] and will be manually assessed for eligibility. In addition, a snowballing technique will be applied by reviewing the reference lists of the included studies to identify additional relevant articles.

### Step 3: study selection

Following the search process, all retrieved literature will be uploaded to Zotero 6 (Center for History and New Media, George Mason University, USA). Duplicate entries will be identified and removed, and the remaining articles are transferred to Rayyan for further screening [66]. For non-English studies, we will engage professional academic translators. These translators will have subject-matter expertise and experience in translating peer-reviewed medical literature. A second qualified translator will review the translations to ensure accuracy and consistency. Two independent reviewers will thoroughly screen the titles and abstracts of the studies using the inclusion and exclusion criteria established for this review.

Two independent reviewers will conduct a detailed assessment of the full texts of the selected articles based on predefined study criteria. If a source does not satisfy the inclusion criteria, the reviewers will record the reason for exclusion. In instances of disagreement during the selection process, the third and fourth reviewers will be consulted to mediate and reach a resolution through discussion [67]. The third and fourth reviewers will refer to the established inclusion and exclusion criteria, along with the recorded reasons for exclusion, to identify and address the underlying causes of disagreement.

Inter-rater reliability between independent reviewers will be assessed using the kappa (κ) statistic [68]. The calculated κ values will be interpreted according to the following established scale: ≤0.20 indicates no agreement, 0.21–0.39 minimal, 0.40–0.59 weak, 0.60–0.79 moderate, 0.80–0.90 strong, and >0.90 almost perfect agreement [68]. A comprehensive summary of the search results and study selection process will be provided in the final scoping review, utilizing the PRISMA 2020 flow diagram for visualization [54].

### Step 4: chart the data/data items

The selected data will be organized and recorded using a custom-designed data extraction tool in Microsoft Excel (Additional file 2: Table 2). Two independent reviewers will conduct this process carefully reviewing each study to extract relevant information.

The extracted data will include the following details: (ⅰ) author, (ⅱ) year of publication, (ⅲ) country of study, (ⅳ) aim, (ⅴ) study design, (ⅵ) study setting, (ⅶ) population and sample size, (ⅷ) participant characteristics, (ⅸ) internal factors that contribute to the improvement of self-management, (ⅹ) findings measures of improvement of self-management, and (ⅺ) findings related to the improvement of self-management. Table 2 presents a preliminary draft that may be refined through iterative discussions during the review process.

The data extracted by the reviewers will be compared for consistency. In the event of any discrepancies, the third and fourth reviewers will participate in discussions to facilitate consensus and maintain uniformity in the data extraction process [67]. If necessary, the authors of the original studies will be contacted to provide the missing data or to clarify specific details. This approach will help to identify research gaps effectively. Given that this review seeks to provide a comprehensive overview of the available evidence, a formal assessment of the risk of bias will not be conducted [54].

### Step 5: collect, summarize, and report the results

This scoping review aims to examine the existing evidence on the internal factors that contribute to the improvement of self-management among T2DM patients during the COVID-19 pandemic and to identify gaps in the current research. To achieve this objective, the extracted data will be systematically organized and categorized based on predefined criteria and presented in tables or graphs. The characteristics and outcomes of the internal factors contributing to the improvement of self-management among T2DM patients during the COVID-19 pandemic will be comprehensively summarized through visual representations, including tables or graphs, and supported by a detailed narrative synthesis of the findings.

### Step 6: consultation

Engaging stakeholders is frequently a valuable component of scoping reviews, as it helps incorporate diverse sources of information and perspectives [69]. In this review, to enhance the clinical relevance and validity of the findings, we will seek feedback on the interpretation of results from certified diabetologists, certified diabetes educators, and certified nurses in diabetes care.

## Discussion

This scoping review aims to systematically map the evidence on internal factors that improve self-management in patients with T2DM during the COVID-19 pandemic and to identify research gaps.

Previous reviews have primarily focused on the external factors supporting T2DM self-management [47, 48] and clinical outcomes during the pandemic [38, 43, 44]. Although evidence of internal factors exists in isolated studies [28, 30, 34, 37, 38], integrated analyses and systematic reviews have not been conducted.

This review holds critical significance in the context of humanitarian emergencies. In such situations, severely restricted access to external resources, including healthcare, makes effective self-management indispensable for preventing fatal outcomes. During the COVID-19 pandemic, for example, when medical resources were severely strained, approximately 40% of deaths occurred in patients with diabetes [70]. Even under these conditions, internal factors serve as direct motivators for self-management [71]. Accordingly, by focusing on these internal factors, this review provides essential insights for informing future crisis responses and clinical interventions.

However, this review has several limitations. First, although it follows the rigorous methodology outlined in the scoping review guidelines of the Joanna Briggs Institute, scoping reviews have inherent limitations. Due to the nature of scoping reviews, it is not possible to determine causal relationships between internal factors and improvements in self-management, nor to identify associations between internal factors and regional or demographic characteristics. Moreover, it is also not feasible to examine interactions or causal links among the internal factors themselves. Additionally, they cannot estimate effect sizes or generate themes based on qualitative analysis. Second, this review does not assess the quality of the included studies or impose restrictions based on study design, which may lead to variability in reliability and generalizability. Third, our search was limited to major international databases, which may have led to the omission of relevant studies indexed exclusively in region-specific databases (e.g., Ichushi-Web for Japanese literature). Fourth, the operational definitions of internal factors, the core concept of this review, may vary across the included studies. For instance, concepts such as self-efficacy and motivation might be defined and measured using different instruments or theoretical frameworks.

These limitations underscore that this review does not present definitive conclusions but rather serves as an exploratory starting point in this research domain. To our knowledge, it is the first review to focus specifically on internal factors that influence T2DM self-management during public health emergencies.

The findings provide a foundation for developing effective self-management strategies and sustainable support systems for T2DM patients, especially in contexts where external support is limited. To translate this impact into clinical, educational, and policy practice, the findings will be strategically disseminated through domestic and international conferences and peer-reviewed publications. Thus, this dissemination is not merely an adjunct but an integral part of the review’s academic value, and it is essential for advancing future research and practice.

## Supplementary Information

Additional file 1: Table 1 Initial search of PubMed (MEDLINE) electronic database.

Additional file 2: Table 2 Data extraction form.

Additional file 3: PRISMA-P (Preferred reporting items for the systematic review and meta-analysis protocol) checklist.

## Data Availability

Not applicable.

## Abbreviations

CINAHL: Cumulative Index to Nursing and Allied Health Literature
COVID-19: Coronavirus disease 2019
ERIC: Education Resources Information Center
HbA1c: Glycated hemoglobin
MeSH: Medical Subject Headings
PRISMA-P: Preferred Reporting Items for Systematic Reviews and Meta-Analyses Protocols
PRISMA-ScR: Preferred Reporting Items for Systematic Reviews and Meta-Analyses for Scoping Reviews
T2DM: Type 2 diabetes mellitus
WHO: World Health Organization
