# Supplementary material for: Internal factors related to self-management among type 2 diabetes patients during the COVID-19 pandemic as humanitarian emergencies: a scoping review protocol
Source: Syst Rev. 2025 Dec 30;14:253. doi: 10.1186/s13643-025-03001-z (PMC12755003; doi:10.1186/s13643-025-03001-z)
Supplement: Supplementary file 1 — Additional file 1: Table 1 Initial search of PubMed (MEDLINE) electronic database. [file 13643_2025_3001_MOESM1_ESM.docx]

Table 1. Initial search in the PubMed (MEDLINE) electronic database

| **No.** | **Query** | **Results** |
| --- | --- | --- |
| #1 | “Type 2 diabetes mellitus”[MeSH] OR “Type 2 diabetes”[TW] OR “T2DM” [TW] | 266,778 |
| #2 | “Self-Management”[MeSH] OR “self-management”[TW] OR “Self-care”[MeSH] OR “self-care”[TW] OR “Self-regulation” [MeSH] OR “self-regulation”[TW] OR “Self-monitoring”[MeSH] OR “self-monitoring”[TW] | 128,367 |
| #3 | (“Resilience, Psychological”[Mesh] OR resilience*[TW]) OR (“self-efficacy”[Mesh] OR “self-efficacy”[TW] OR “self efficacy”[TW] OR “self-efficac*”[TW]) OR (“diabetes distress”[MeSH] OR “diabetes distress”[TW] OR “diabetes-related distress”[TW] OR “diabetes emotional distress”[TW]) OR (“meaningfulness of life”[Mesh] OR “meaningfulness of life”[TW] OR meaningful*[TW]) OR (“self-confidence”[Mesh] OR “self-confidence”[TW] OR “self confidence”[TW] OR self-confidence*[TW] OR self confidence*[TW]) OR (“health literacy”[Mesh] OR “health literacy”[TW] OR “health literacies”[TW] OR “health literac*”[TW]) OR (“stress management”[Mesh] OR “stress management”[TW] OR stress*[TW] OR “stress coping”[TW] OR “stress reduction”[TW])  OR (“Coping Skills”[Mesh] OR “coping skill*”[TW]) OR (“problem-solving ability”[Mesh] OR “problem-solving ability”[TW] OR “problem solving”[TW] OR “problem-solving”[TW]) OR (“motivation”[Mesh] OR motivation*[TW]) OR (“Decision Making”[Mesh] OR “decision-making ability”[TW] OR “decision making ability”[TW] OR “decision-making”[TW] OR “decision making”[TW]) OR (“self-control”[Mesh] OR “self-control”[TW] OR self control[TW] OR self-control*[TW]) OR (“illness belief”[Mesh] OR “illness belief*”[TW]) OR (“health belief”[Mesh] OR “health belief*”[TW]) | 2,462,607 |
| #4 | (“COVID-19 pandemic”[MeSH] OR “COVID-19 pandemic”[TW] OR “COVID-19”[TW]) AND (“pandemic”[TW] OR “epidemic”[TW]) | 236,426 |
| #5 | (“maintenance"[MeSH] OR maintenance*[TW]) OR (“Improvement” [MeSH] OR “improv*” [TW]) OR (“Effectiveness” [MeSH] OR “effect*” [TW]) OR (“Enhancement” [MeSH] OR “enhance*” [TW]) | 15,679,205 |
| #6 | #1 AND #2 OR #3 AND #4 AND #5 | 22,376 |
| #7 | #1 AND #2 OR #3 AND #4 AND #5 (“2020/03/11” [PDAT]: “3000” [PDAT]) | 22,274 |
