# Supplementary material for: Internal factors related to self-management among type 2 diabetes patients during the COVID-19 pandemic as humanitarian emergencies: a scoping review protocol
Source: Syst Rev. 2025 Dec 30;14:253. doi: 10.1186/s13643-025-03001-z (PMC12755003; doi:10.1186/s13643-025-03001-z)
Supplement: Supplementary file 2 — Additional file 2: Table 2 Data extraction form. [file 13643_2025_3001_MOESM2_ESM.docx]

Table 2. Data extraction form

| Categories | Type of data |
| --- | --- |
| 1. basic characteristics | author |
|  | year of publication |
|  | country of study |
|  | aim |
|  | study design |
|  | population and sample size  data collection period (early, middle, and late stages of the COVID-19 pandemic )  mean age |
| 2. Participants characteristics | type 2 diabetes duration  treatment for type 2 diabetes (oral hypoglycemic agents, self-administered insulin injection)  sex  Presence or absence of complications (e.g., diabetic nephropathy, retinopathy, peripheral neuropathy)  Place of residence Educational level Employment status and economic condition |
| 3. Internal factors | resilience, self-efficacy, diabetes distress, meaningfulness of life, self-confidence, health literacy, stress management behavior and coping skills, problem-solving ability, motivation, decision-making ability, self-control, illness belief and health belief |
| 4. finding measures | structured Questionnaires  interviews  observational data |
| 5. findings related to maintenance and improvement | behavioral logs or diaries  physiological or biological measures (e.g., blood glucose level, HbA1c, blood pressure, weight) Patient-Reported Outcomes |
